# Among the Colleges

**Published:** 1895-12

**Authors:** 


### AMONG THE COLLEGES.

---

Two of the English veterinary colleges will rearrange their sessions so as to have an interval of a month in winter and six weeks in the spring, completing the course by a summer session. This will afford students the opportunity of securing practical opportunities with their preceptors at those seasons of the year when the average practitioner is generally very busy, and at a time when specialties of practice are seen to a greater extent.

THE INDIANA VETERINARY COLLEGE held its fourth annual commencement exercises in the lecture-room in connection with the college on the afternoon of the 17th day of March, 1895.

Opening remarks were made by the President, H. Armstrong. This was followed by short, appropriate remarks by others of the faculty, at the conclusion of which the degree of V.S. was conferred upon the following students: Louis A. Grenier, Indianapolis, Ind. ; J. B. Heaton, Bloomfield, Ind. ; Claude Wilson, Greenfield, Ind. ; J. B. Cox, Carlyle, Ind. ; Samuel Rodebaugh, New Augusta, Ind.

VETERINARY DEPARTMENT, IOWA AGRICULUTRAL AND MECHANICAL COLLEGE.—The following-named students of the above college have received the degree of D.V.M. : A. W. Bitting, Lafayette, Ind. ; Richard Blanche, Conrad, Ia. ; E. T. Davidson, Burt, Ia. ; L. L. Lewis, Rhea's Mills, Texas ; F. S. Roop, Childress, Va. ; and F. L. Rice, Decorah, Ia.

